# Short Dark Tetrad - Polish version (SD4-PL) - examination of psychopathy, narcissism, Machiavellianism and sadism - preliminary psychometric properties of the tool in Polish conditions

**DOI:** 10.1192/j.eurpsy.2025.1914

**Published:** 2025-08-26

**Authors:** P. Dębski, P. Pałczyński, M. Garczarczyk, M. Piankowska, K. Haratyk, M. Noras, M. Szostak, M. Meisner

**Affiliations:** 1 Department of Psychiatry, Faculty of Medical Sciences in Zabrze, Medical University of Silesia, Katowice; 2 Institute of Psychology, Faculty of Social Sciences and Humanities, Humanitas University, Sosnowiec, Poland

## Abstract

**Introduction:**

The Dark Tetrad of Personality includes factors commonly recognized as socially aversive. So far, they have been recognized as psychopathy, narcissism and Machiavellianism. Nowadays, sadism has also been included in the scope of dark personality traits. There are many tools for examining the Dark Triad of Personality, while tools dedicated to the Dark Tetrad are under development. One such tool is The Short Dark Tetrad scale (SD4) by Paulhus et al. (2021), which, due to its popularity, may enable cross-cultural research in the future.

**Objectives:**

The aim of the study was to assess the preliminary psychometric properties of the Short Dark Tetrad tool - Polish version (SD4-PL).

**Methods:**

972 people with an average age of 34.39±11.09 SD years were recruited for the study. The 28-item Short Dark Tetrad scale (SD4) was translated into Polish (SD4 Pl) and used for a study. As part of the statistical analyses, a confirmatory factor analysis (CFA) was used, based on the structural equation modeling. The internal consistency of the tool was also assessed.

**Results:**

Confirmatory factor analysis (CFA) parameters turned out to be satisfactory. The original structure of the tool was maintained, which consists of four 7-item scales dedicated to psychopathy, narcissism, Machiavellianism and sadism. The obtained psychometric parameters turned out to be similar to the results obtained in several countries where the Short Dark Tetrad scale has already been introduced. Satisfactory measures confirming the reliability of the tool (Cronbach’s α) were also obtained, ranging between 0.68 and 0.79.

**Conclusions:**

Short Dark Tetrad - Polish version (SD4-PL) presents satisfactory psychometric properties. By maintaining the original structure of the tool, it opens the possibility of examining its equivalence with other language versions and, in the future, the possibility of conducting international research focusing on factors such as psychopathy, narcissism, Machiavellianism and sadism.

**Disclosure of Interest:**

None Declared

